# Fecal Microbiota Transplantation from Methionine-Restricted Diet Mouse Donors Improves Alzheimer’s Learning and Memory Abilities Through Short-Chain Fatty Acids

**DOI:** 10.3390/foods14010101

**Published:** 2025-01-02

**Authors:** Run Yu, Haimeng Zhang, Rui Chen, Yangzhuo Lin, Jingxuan Xu, Ziyang Fang, Yuehang Ru, Chenhan Fan, Guoqing Wu

**Affiliations:** 1 School of Public Health, Health Science Center, Ningbo University, Ningbo 315211, China; 2 School of Basic Medical Science, Health Science Center, Ningbo University, Ningbo 315211, China

**Keywords:** fecal microbiota transplantation, methionine restriction, SCFAs, Alzheimer’s disease

## Abstract

Alzheimer’s disease (AD) is marked by impaired cognitive functions, particularly in learning and memory, owing to complex and diverse mechanisms. Methionine restriction (MR) has been found to exert a mitigating effect on brain oxidative stress to improve AD. However, the bidirectional crosstalk between the gut and brain through which MR enhances learning and memory in AD, as well as the effects of fecal microbiota transplantation (FMT) from MR mice on AD mice, remains underexplored. In this study, APP/PS1 double transgenic AD mice were used and an FMT experiment was conducted. 16S rRNA gene sequencing, targeted metabolomics, and microbial metabolite short-chain fatty acids (SCFAs) of feces samples were analyzed. The results showed that MR reversed the reduction in SCFAs induced by AD, and further activated the free fatty acid receptors, FFAR2 and FFAR3, as well as the transport protein MCT1, thereby signaling to the brain to mitigate inflammation and enhance the learning and memory capabilities. Furthermore, the FMT experiment from methionine-restricted diet mouse donors showed that mice receiving FMT ameliorated Alzheimer’s learning and memory ability through SCFAs. This study offers novel non-pharmaceutical intervention strategies for AD prevention.

## 1. Introduction

Dementia is one of the most pressing global challenges, with Alzheimer’s disease (AD) being its most prevalent form. The interactions among various mechanisms underlying AD are complex and multi-layered, posing significant challenges for developing effective treatment strategies for AD. To date, AD cannot be completely cured, and non-pharmacological interventions are believed to prevent or delay AD [1]. A diet rich in fruits, vegetables, and legumes, while low in meat and sugar, is considered beneficial for reducing the risk of AD [2]. Methionine, an amino acid abundant in meat—particularly red meat—is present in much lower quantities in nuts, legumes, grains, and pasta, and is almost absent in fruits and vegetables [3]. Therefore, a diet with methionine restriction (MR), which limits methionine intake to 20% of the recommended amount and closely resembles a vegetarian diet [3], is theoretically considered, in that an MR diet holds promise to mitigate the risk of AD.

Moreover, numerous studies have demonstrated that MR can significantly reduce white adipose tissue content, prevent obesity and type 2 diabetes, inhibit cancer development, delay aging processes, and extend the lifespan [4,5,6]. Furthermore, our previous research indicated that MR intervention in middle-aged mice exhibited enhanced anti-depressive and learning memory capabilities [6]. In D-galactose-induced aging mice, MR can lower the levels of Aβ and enhance the learning and memory function [7]. Another study reported a significant positive correlation between a high dietary intake of methionine and mild cognitive impairment risk in individuals [8]. The study revealed that MR can mitigate brain oxidative stress via the CBS/H_2_S pathway to relieve the learning and memory deficits in AD mouse models [8]. However, in AD mice, studies on the bidirectional crosstalk between the gut and brain through which MR improves Alzheimer’s learning and memory ability are limited. Given the intricate nature of AD pathology, further investigation is essential to elucidate the gut–brain regulatory mechanisms underlying the cognitive benefits of MR in AD.

The close correlation between AD and an imbalanced gut microbiota has been supported by multiple lines of evidence [9,10]. The impact of the “microbiota-gut-brain axis” on AD has attracted much attention [11]. Important metabolites of gut microbiota, such as short-chain fatty acids (SCFAs), can regulate microglia activation and transformation, relieve cerebral inflammation and Aβ accumulation, and alleviate behavioral disorders in AD mice [12,13]. Moreover, recent research has showed that MR contributed to a potential role in cognitive dysfunction in 8-week-old ICR mice via the dose-dependent inhibition of SCFAs [14]. However, in AD mice, the direct regulatory effect of MR on the gut microbiota or SCFAs and their impacts on learning and memory ability through the “microbiota-gut-brain axis” remains unexplored.

Fecal microbiota transplantation (FMT) from normal mice has been shown to mitigate the development of AD in mice [15]. Therefore, enriching or reconstructing the gut microbiota intervention strategies have become feasible choices for preventing and managing AD. In AD mice, the potential role of FMT from methionine-restricted diet mouse donors in ameliorating Alzheimer’s learning and memory ability through the gut–brain axis remains to be explored. Hence, the objective of this present study is to research whether both MR intervention and FMT from methionine-restricted diet mice donors can enhance the cognitive function and memory by modulating the gut microbiota or SCFA levels in AD mice, thereby achieving a preventive effect on Alzheimer’s disease. This study will provide new strategies for non-pharmaceutical intervention to prevent AD.

## 2. Materials and Methods

### 2.1. Animals

All animal studies were approved by the Laboratory Animals Ethics Committee of Ningbo University (permission number NBU20210034) and conducted in accordance with the Chinese guidelines for animal welfare and experimental protocol after approval by the Animal Care and Use Committee of the Animal Nutrition Institute of Ningbo University. APP/PS1 mice and littermate wild-type mice were purchased from Hangzhou Ziyuan experimental animal Technology Co., Ltd. (Hangzhou, China). The mice were housed in standard environmental conditions and provided with ad libitum access to water and food throughout the study.

### 2.2. Dosage Regimen

**Figure 1:**
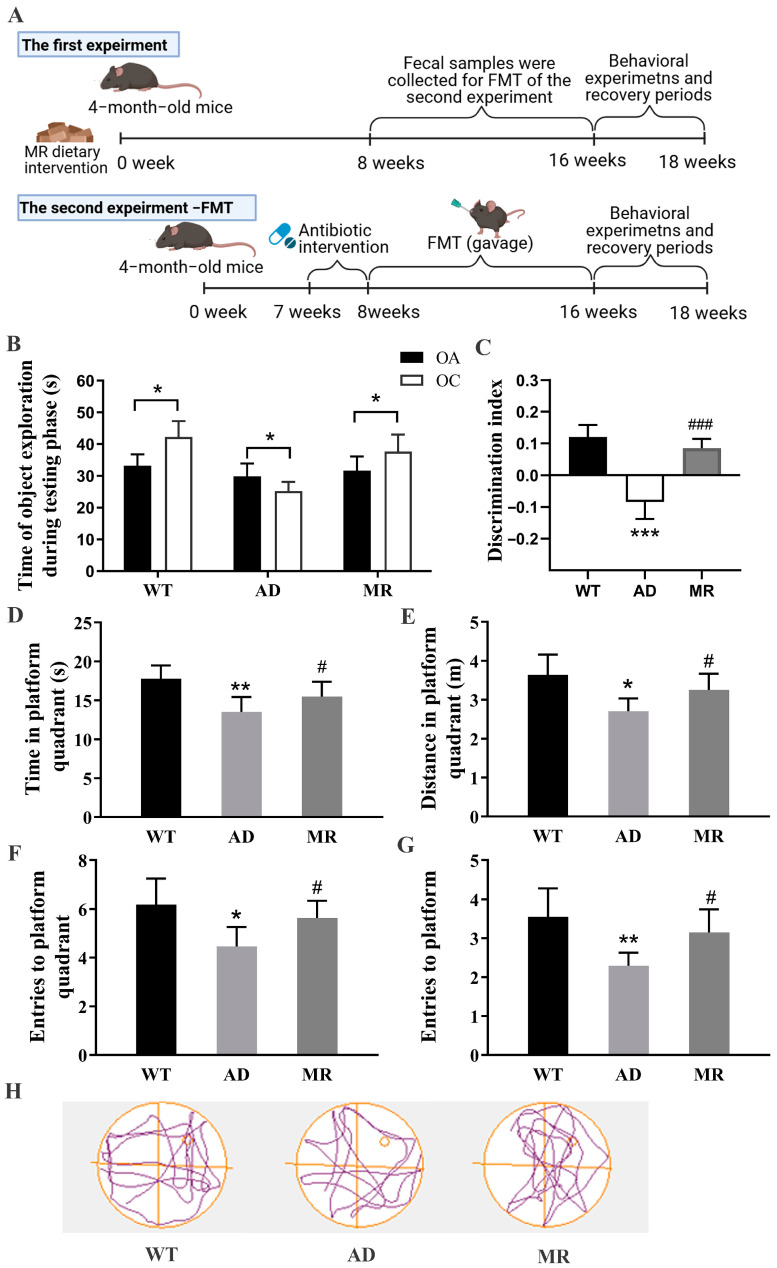
Experimental design of two experiments and the effects of MR intervention on learning and memory abilities in AD mice. (**A**) Experimental design of two experiments including MR intervention in AD mice experiment and fecal microbiota transplantation (FMT) experiment. (**B**,**C**) MR ameliorated nonspatial recognition memory ability of AD mice in the novel object recognition test, including (**B**) exploration time in the test phase and (**C**) discrimination index. (**D**–**H**) MR ameliorated spatial memory ability of AD mice in the Morris water maze test, including (**D**) time in the platform quadrant, (**E**) distance in the platform quadrant, (**F**) entries to the platform quadrant, (**G**) entries to the platform, and (**H**) track plot. Values are presented as means ± SEMs (n = 10 per group). *, *p* < 0.05; **, *p* < 0.01; ***, *p* < 0.001; AD vs. WT. #, *p* < 0.05; ###, *p* < 0.001; MR vs. AD.

The experimental design is illustrated in Figure 1A. In the first experiment, twenty male APP/PS1 mice (4.5 months old) and ten littermate wild-type mice were divided into three groups: (1) WT group, wild-type mice with a standard diet (n = 10, 0.86% methionine), (2) AD group, APP/PS1 mice with a standard diet (n = 10, 0.86% methionine), and (3) MR group, APP/PS1 mice with a MR diet (n = 10, 0.17% methionine). The period of this experiment was 18 consecutive weeks. In the second experiment (FMT experiment), thirty male APP/PS1 mice (3.5 months old) were divided into three groups: (1) AD group, APP/PS1 mice were fed a standard diet (n = 10), (2) AA group, APP/PS1 mice were fed a standard diet and transplanted fecal microbiota from the AD groups of the first experiment (n = 10), and (3) MA group, APP/PS1 mice were fed a standard control diet and transplanted fecal microbiota from the MR groups of the first experiment (n = 10). The period of this experiment was 13 consecutive weeks. Additionally, the MR diet was designed according to a previous study [16]. The feed was purchased from Dyets Biotechnology (Wuxi) Ltd., Wuxi, China.

### 2.3. Novel Object Recognition Test and the Morris Water Maze Test

As classic behavioral experiments, the novel object recognition test (NORT) and Morris water maze (MWM) test were used to assess the non-spatial and the spatial declarative memory, respectively, as previously reported [17,18]. The detailed steps are provided in the Supplementary Materials.

### 2.4. Tissue and Plasma Preparation

After a fasting period of 8–12 h, the mice were administered pentobarbital sodium (50 mg/kg body weight, i.p.) to induce anesthesia. Blood was collected from the retro-orbital sinus plexus into tubes containing a plasma separator. Plasma was then obtained through centrifugation. Immediately following blood collection, the animal care technicians performed dissections of tissues, including the brain, hippocampus, and ileum.

### 2.5. Histological Analysis and Statistical Calculation

The ileum samples were preserved in 10% formaldehyde phosphate buffer, dehydrated, and embedded in paraffin. Thin sections measuring 5–8 μm were prepared, mounted on slides, and stained with hematoxylin and eosin. The sections were then examined under a CX31 RTSF microscope and quantified using Image-Pro Plus 6.0 software.

### 2.6. Brain Biochemical Indices

The brain inflammatory biomarkers of interleukin-1β (IL-1β) and tumor necrosis factor-alpha (TNF-α) were measured using the ELISA kits (Hangzhou Lianke Biotechnology Co., Ltd., Hangzhou, China).

### 2.7. Total RNA Extraction and Quantitative RT-PCR (qRT-PCR)

The total RNA was extracted from the brain tissue using Trizol reagent and quantified using a NanoDrop Spectrophotometer (ND2000, Thermo, Waltham, MA, USA). A PrimeScript RT reagent Kit with gDNA Eraser (Takara Bio USA, Inc., Mountain View, CA, USA) was used in a 7900HT instrument (Applied Biosystems, Forster, CA, USA). The gene sequence of BDNF was the forward primer CGATTAGGTGGCTTCATAGGAGAC and reverse primer CAGAACAGAACAGAACAGAACAGG. The gene sequence of GDNF was the forward primer AGAGGGGCAAAAATCGGG and reverse primer CCGCTGCAATATCGAAAGATCA. The gene sequence of Occludin was the forward primer GTGTGGTTGATCCCCAGGAG and reverse primer TCGCTTGCCATTCACTTTGC. The gene sequence of Claudin-2 was the forward primer CCCAGGCCATGATGGTGA and reverse primer TCATGCCCACCACAGAGATAAT. The gene sequence of ZO-1 was theforward primer TACCTCTTGAGCCTTGAACTT and reverse primer CGTGCTGATGTGCCATAATA. The gene sequence of β-actin was the forward primer GGCTGTATTCCCCTCCATCG and reverse primer CCAGTTGGTAACAATGCCATGT. These gene primers were synthesized by Sangon Biotech (Shanghai, China) Co., Ltd.

### 2.8. Measurement of SCFAs

A total of 1000 µL of 0.005 M aqueous NaOH containing an internal standard (5 µg mL^−1^ caproic acid-d3) was added to the feces samples (50–150 mg), and the samples were homogenized and centrifuged individually. Then the samples were derived, and the derivatives were extracted via a two-step extraction using hexane, and analyzed using GC-MS. The detailed protocols were produced as previously described [19]. The standards of SCFAs (acetic acid, butyric acid, and propionic acid) and internal standard d3-caproic acid were all purchased from Aladdin (Shanghai, China).

### 2.9. Targeted Metabolomics and 16S Ribosomal RNA Gene Sequencing

The targeted metabolomics analysis of the fecal samples was conducted by Wuhan Metware Metabolic Biotechnology Co., Ltd. (Wuhan, China). Additionally, 16S ribosomal RNA gene sequencing of the fecal samples was performed by Biomarker Technologies Co., Ltd. (Beijing, China). The detailed sequencing and analytical procedures are provided in the Supplementary Materials.

### 2.10. Fecal Microbiota Transplantation

Before the FMT treatment, the mice were administered a combination of four antibiotics (1 g/L ampicillin, 1 g/L neomycin, 1 g/L metronidazole, and 0.5 g/L vancomycin) in their drinking water at a dosage of 0.15 mL/mouse daily for one week to eliminate the indigenous gut microbiota, as previously described with a modification [20,21]. Then, the steps of gut microbial transplantation were described as per previous research [22]. Fresh gut microbiota from the donor mice was obtained through suspending the gathered fecal samples in sterilized PBS (50 mg of the sample with the addition of 1 mL of PBS) followed by centrifugation (1000 rcf). After seven days of antibiotic water interference, the microbial gavage intervention was administered for a duration of 8 weeks, following a schedule of 3 consecutive days followed by 1 day of rest in the AA and MA groups.

### 2.11. Statistical Analyses

The bioinformatics analysis for this study was conducted using the BMK Cloud platform (Biomarker Technologies Co., Ltd., Beijing, China). The statistical significance was evaluated using one-way ANOVA followed by Tukey’s test, performed using SPSS 22.0 software. A *p*-value of less than 0.05 was considered statistically significant.

## 3. Results

### 3.1. MR Improved the Cognitive Ability of AD Mice

The NORT and MWM test were used to exam the cognitive ability. The NORT results revealed that, in the test phase, both the AD mice fed with MR and WT mice demonstrated a noticeably higher inclination towards exploring a novel object (OC) compared to a familiar object (OA). In contrast, untreated AD mice exhibited a preference for the familiar object (*p* < 0.05, Figure 1B). Additionally, compared with the WT mice, in the AD mice, the discrimination index was significantly reduced, but MR treatment significantly improved this index (*p* < 0.001, Figure 1C). These findings indicated that MR intervention enhanced the non-spatial memory in AD mice.

Spatial learning and memory were further evaluated using the MWM test. In the probe trial task of MWM, time or distance in the platform quadrant, the number of entries to the platform quadrant, and entries to the platform all declined in the AD group, and these levels were significantly raised via MR (*p* < 0.05 or *p* < 0.01, Figure 1D–H). Compared with that of the AD groups, the tracking plots of the WT and MR groups were evidently concentrated in the targeted platform quadrant (Figure 1H). These results indicated that MR intervention enhanced the spatial memory in AD mice.

### 3.2. MR Improved the Gut Microbiota

**Figure 2:**
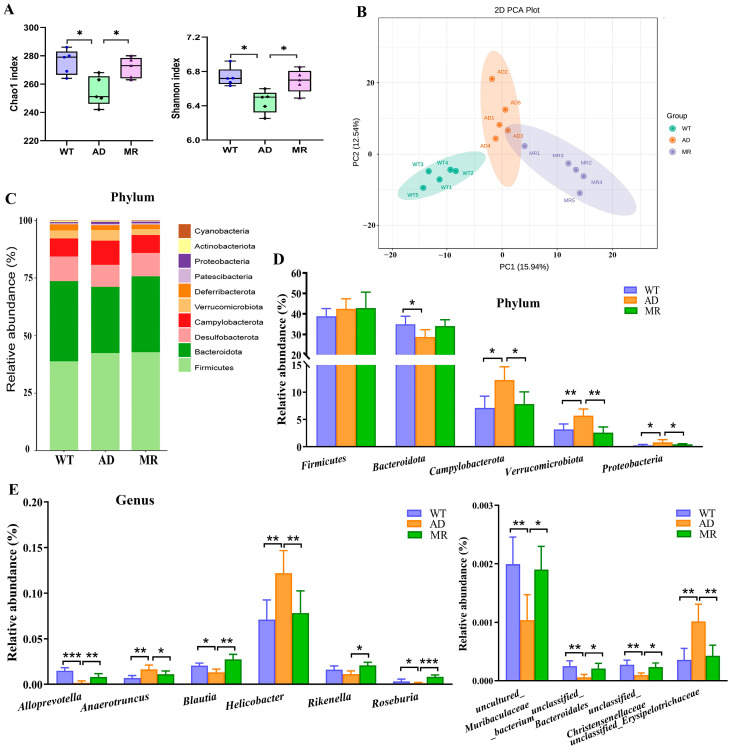
(**A**) Alpha-diversity indexes: Chao1 and Shannon indexes. (**B**) Beta diversity estimated via principal component analysis (PCA). (**C**) Stack diagram of the variation in fecal microbiota at the phylum level. (**D**) Comparison of relative abundance at the phylum level using the ANOVA biomarker. (**E**) Comparison of relative abundance at genus level. Values are presented as means ± SEMs (n = 5 per group). *, *p* < 0.05; **, *p* < 0.01; ***, *p* < 0.001.

Alpha-diversity and beta-diversity indices were used to estimate the bacterial diversity and richness (Figure 2A,B). The results of the first experiment showed that the AD mice had notably low Chao1 and Shannon indexes compared to that in the WT mice (*p* < 0.05), and MR increased these two indexes (*p* < 0.05). According to the beta-diversity index, the scatter plot of PCA exhibited a significant separation of the fecal microbiota among each group (*p* < 0.05, Figure 2B). These results indicated that MR could improve the reduction in bacterial diversity caused by AD.

At the phylum level, the AD mice significantly reduced the *Bacteroidota* abundance. Moreover, AD remarkably increased the *Campylobacerota*, *Verrucomicrobia*, and *Proteobacteria* abundances while MR treatment significantly reduced their levels (*p* < 0.05, Figure 2C,D). Several reports about 9-month-old and 12-month-old APP/PS1 mice showed that although the results for *Firmicutes* and *Bacteroidetes* were inconsistent, the reduction in *Proteobacteria* and *Verrucobacteria* was a common feature [23,24]. At the genus level, compared with the WT group, the AD mice experienced a distinct increase in the abundance of *Anaerotruncus*, *Helicobacter*, and *unclassified Erysipelotrichaceae* (*p* < 0.01, Figure 2E), which is consistent with the literature reports [23,25]. MR significantly reduced the levels of these harmful bacteria. Moreover, the AD group had a significant drop in the abundance of *Alloprevotella* (*p* < 0.001), *Blautia* (*p* < 0.05), *Roseburia* (*p* < 0.05), *uncultured Muribaculaceae bacterium* (*p* < 0.01), *unclassified Bacteroidales*, and *unclassified Christensenellaceae* (Figure 2E). The reductions in *Alloprevotel*, *Blautia*, *Roseburia*, and *Bacteroidales* abundances due to AD were reported in previous reports [23,26,27,28], while MR significantly increased these six beneficial bacteria and the good bacterium level of *Rikenella* (*p* < 0.05, Figure 2E).

### 3.3. MR Enhanced the Mucosal Barrier Function

**Figure 3:**
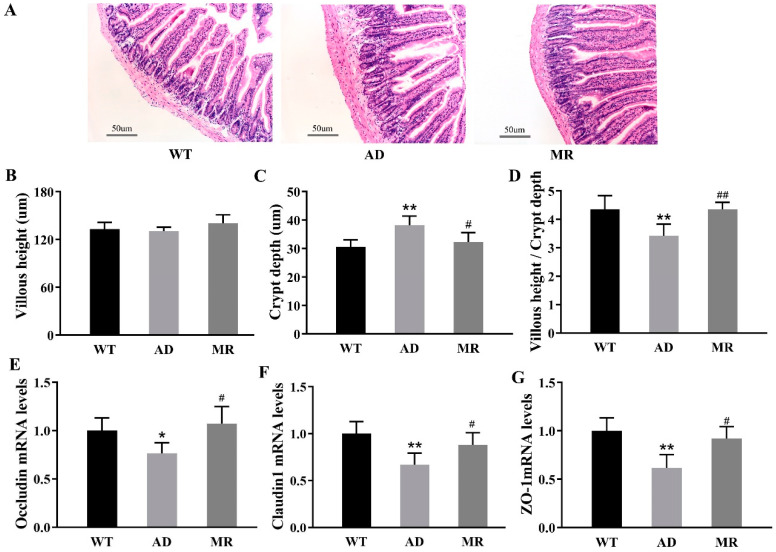
MR enhanced mucosal barrier function. (**A**) Hematoxylin and eosin (H&E) staining of ileal sections (200×). (**B**–**D**) Histological analysis. (**E**–**G**) Intestinal mucosal immunity barrier indexes: gene expression levels of Occludin, Claudin1, and ZO-1 in the ileum. Values are presented as means ± SEMs (n = 6 per group). *, *p* < 0.05; **, *p* < 0.01; AD vs. WT. #, *p* < 0.05; ##, *p* < 0.01; MR vs. AD.

Based on the hematoxylin and eosin staining of ileal sections (Figure 3A), the villous height, crypt depth, and the ratio of villous height to crypt depth were calculated (Figure 3B–D). Compared to the WT group, the AD mice had a significant increase in crypt depth, resulting in a decrease in the ratio of villous height to crypt depth (*p* < 0.01), while MR normalized these indexes (*p* < 0.05 and *p* < 0.01, Figure 3B–D). Moreover, compared to the AD group, in the MR group, the gene expression of the ileal membrane-spanning proteins Occludin and Claudin1, and the tight junction protein 1(ZO-1) was significantly increased (*p* < 0.05, Figure 3E–G).

### 3.4. MR Improves the Fecal Metabolites

**Figure 4:**
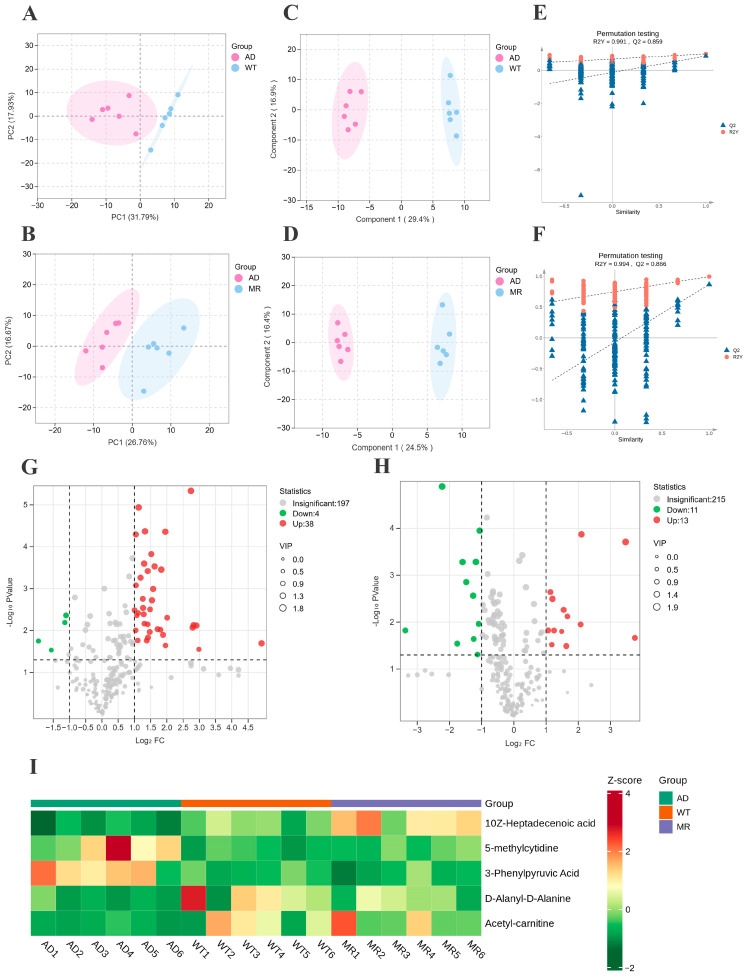
Metabolomics analysis of fecal samples. (**A**,**B**) PCA score plot (AD vs. WT and MR vs. AD) (**C**,**D**) OPLS-DA scores (AD vs. WT and MR vs. AD). (**E**,**F**) Permutation test of the OPLS-DA model (AD vs. WT and MR vs. AD). (**G**,**H**) Volcano plot illustrating the differential expression of metabolites between (AD vs. WT and MR vs. AD). (**I**) Heatmap depicting significantly different fecal metabolites among the three groups.

The targeted metabolomics of feces, the key component in the gut–brain axis, can significantly contribute to a deeper understanding of the mechanism through which MR improves AD through the modulation of the gut microbiota. The score plot of principal component analysis (PCA) (Figure 4A,B) clearly demonstrates a distinct separation between the AD group and CON group, as well as between the MR group and AD group, indicating significant disparities in the metabolites and indirectly suggesting metabolic disorders in AD mice. The fecal samples from the three groups were further analyzed using orthogonal partial least squares discriminant analysis (OPLS-DA) to identify the differential metabolites. The OPLS-DA score plot clearly demonstrates a distinct separation between the AD group and both the CON group and MR group (Figure 4C,D). The validity of the OPLS-DA model was determined based on 200 response permutation tests (Figure 4E,F). The R2Y and Q2 values for the control group and AD group were calculated as R2Y = 0.991 and Q2 = 0.659, respectively. Similarly, for the AD and MR groups, the corresponding R2Y and Q2 values were R2Y = 0.994 and Q2 = 0.866, respectively. Both R2Y values approached unity and both Q2 values exceeded 0.5, indicating a robust fit of the OPLS-DA model to fecal samples with excellent predictive capability.

The OPLS-DA results were integrated with the corresponding VIP values of metabolites to identify and extract the intergroup metabolites. Compared with the CON group, a total of 42 distinct metabolites were identified in the AD group, with the levels of four metabolites significantly reduced and the levels of 38 metabolites significantly elevated (Figure 4G). In the MR group, 24 distinct metabolites were identified, which exhibited significant decreases in the levels of 11 metabolites and significant increases in the levels of 13 metabolites when compared to the AD group (Figure 4H).

To further analyze the differential metabolites, five common metabolites were identified: acetyl-carnitine, 10Z-heptadecenoic acid, D-alanyl-D-alanine, 3-phenylpyruvic acid, and 5-methylcytidine (5 mC) (Figure 4I). These metabolites predominantly reflect oxidative stress, energy metabolism, methylation reactions, and phenylalanine metabolism. Moreover, they are closely associated with the intestinal microbiota. The results depicted in Figure 4I show a significant decrease in the fecal levels of acetyl-carnitine, 10Z-heptadecenoic acid, and D-alanyl-D-alanine among the mice in the AD group compared with those in the CON group. Conversely, the administration of MR significantly elevated the concentrations of these substances in the feces of the AD mice. Acetyl-carnitine, an endogenous metabolic intermediate, possesses antioxidant properties and could effectively reduce amyloid-induced protein and lipid oxidation. Additionally, it regulates the mitochondrial function to prevent ATP depletion [29,30]. Furthermore, it alleviates intestinal inflammation by decreasing the release of inflammatory factors, modulating the ratio of immune cells, and regulating the intestinal immunity [31]. 10Z-Heptadecenoic acid, a monounsaturated fatty acid, serves as a carbon source for β-oxidation in brewing yeast [32], and provides energy while also exhibiting inhibitory effects on the growth of streptococcus mutans. Furthermore, unsaturated fatty acids have been shown to mitigate the cytotoxicity associated with amyloid protein aggregation [33]. As a dipeptide, D-Alanyl-D-Alanine plays a pivotal role in the normal growth of bacterial cells by serving as an essential component of their cell wall [34]. According to these findings, MR might alleviate the symptoms of AD and reduce the bodily damage by enhancing the oxidative stress management, energy metabolism, and unsaturated fatty acid levels, which promotes intestinal bacteria growth, improves the gut microbiota, and protects the gut barrier function.

The data presented in Figure 4I demonstrate that the AD group of mice exhibited elevated levels of 3-Phenylpyruvic acid and 5 mC in their feces, as compared to those in the CON group. However, the administration of MR significantly attenuated these substances’ concentrations in the fecal samples of AD mice. 3-Phenylpyruvic acid is a secondary metabolite of phenylalanine, which can cause DNA damage in glial cells and induce the generation of reactive oxygen species [35], thereby reducing the production of reduced coenzyme II [36]. As a marker of DNA methylation, 5 mC was considered one of the epigenetic modifications associated with the various types of AD. Meanwhile, serine, an essential amino acid involved in intracellular methylation reactions, was closely correlated with AD. It can be inferred that MR improved AD by regulating the body’s redox balance and targeting the methylation metabolism of S-adenosylmethionine and S-adenosylhomocysteine in the methionine pathway.

### 3.5. MR Increased the Concentration of SCFAs and Relative Receptors

According to the gut microbiota composition shown in Figure 2, as determined via 16S ribosomal RNA gene sequencing, the abundances of *Roseburia*, *Bacteroidales*, and *Christensenellaceae* were increased by MR in the AD mice. These bacteria are capable of producing SCFAs, particularly butyrate [28,37,38]. Moreover, the targeted metabolomics analysis of feces (Figure 4) revealed that energy metabolism and REDOX are important regulatory mechanisms in MR. It has been shown that SCFAs regulate the host health by regulating the oxidative stress-inflammatory signals in the intestine and the whole body. SCFAs can be used as an energy source to provide energy for intestinal cells through oxidative decomposition through the tricarboxylic acid cycle. SCFAs can also modulate cerebral substance metabolism, as well as neurological and immunological aspects [39,40].

**Figure 5:**
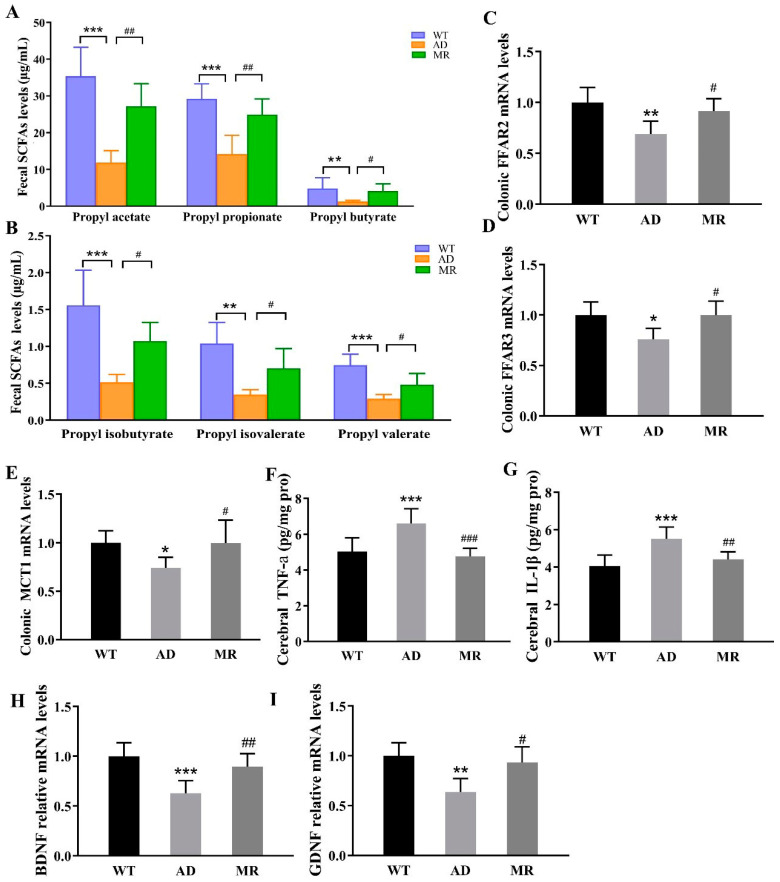
MR increased the levels of SCFAs, free fatty acid receptors, and SCFAs transporters, and improved inflammatory factors, learning, and memory indicators of brain. (**A**,**B**) The concentrations of several types of SCFAs. (**C**,**D**) Free fatty acid receptors FFAR2 and FFAR3. (**E**) SCFA transporter—monocarboxylate transporter (MCT1). (**F**,**G**) Inflammatory factors TNF-a and IL-1β in the brain. (**H**,**I**) The mRNA levels of learning and memory indicators in the hippocampus: brain-derived neurotrophic factor (BDNF) and glial cell line-derived neurotrophic factor (GDNF). Values are presented as means ± SEMs (n = 6 per group). *, *p* < 0.05; **, *p* < 0.01; ***, *p* < 0.001; AD vs. WT. #, *p* < 0.05; ##, *p* < 0.01; ###, *p* < 0.001; MR vs. AD.

The concentration of SCFAs was further tested (Figure 5A,B). Compared to the WT mice, the AD mice experienced a significant decline in the levels of propyl acetate, propyl propionate, propyl butyrate, propyl isobutyrate, propyl isovalerate, and propyl valerate (*p* < 0.01 or *p* < 0.001). MR intervention significantly increased the levels of these metabolites (*p* < 0.05 or *p* < 0.01, Figure 5A,B). These results provide direct evidence of the efficacy of MR in enhancing SCFA production. Moreover, SCFAs activated vagal afferents via the free fatty acid receptors, FFAR2 or FFAR3, thereby signaling to the brain [41]. SCFAs can cross the blood–brain barrier to affect the central nervous system (CNS), possibly facilitated by the abundant expression of MCTs on endothelial cells [41,42]. As shown in Figure 5C–E, MR administration mitigated the AD-induced decline in the gene expression of FFAR2, FFAR3, and MCT1 (*p* < 0.05, Figure 5C–E).

### 3.6. MR Reduced Cerebral Inflammation and Enhanced the Learning and Memory Indexes

SCFAs can improve the reduction in inflammation, behavioral disorders, and Aβ accumulation in AD mice [12,13]. As shown in Figure 5F,G, MR alleviated AD-induced inflammation through reducing the levels of inflammatory factors TNF-a and IL-1β in the brain (*p* < 0.01 and *p* < 0.001). Moreover, AD decreased the expression of learning and memory indicators in the hippocampus including brain-derived neurotrophic factor (BDNF) and glial cell line-derived neurotrophic factor (GDNF), and MR remarkably increased their gene expressions (*p* < 0.05 or *p* < 0.01, Figure 5H,I).

### 3.7. FMT Intervention Improved the Learning and Memory Capacities

**Figure 6:**
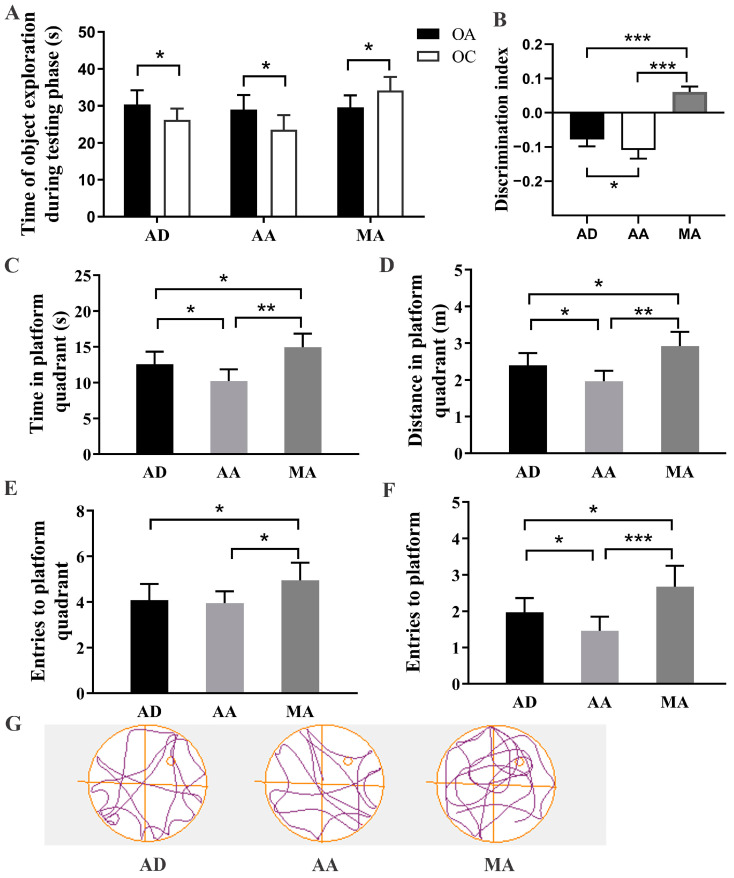
Fecal microbiota transplantation experiment demonstrated the improvement via MR of the cognitive ability of AD mice. Novel object recognition test was used to assess nonspatial recognition memory ability: (**A**) exploration time in the test phase, (**B**) discrimination index. Morris water maze test was used to assess the spatial memory: (**C**) time in the platform quadrant; (**D**) distance in the platform quadrant; (**E**) entries into the platform quadrant; (**F**) entries into the platform; (**G**) track plot. Values are presented as means ± SEMs (n = 10 per group). *, *p* < 0.05; **, *p* < 0.01; ***, *p* < 0.001.

As shown in Figure 6, the NORT results showed that the AD group and AA group preferred older objects (OA), while MA exhibited a significantly greater preference for exploring a novel object (OC) (*p* < 0.05, Figure 6A). Moreover, compared with the AD group, the discrimination index of the AA group was significantly decreased, and MA significantly augmented the discrimination index (*p* < 0.05 and *p* < 0.001, respectively, Figure 6B). Additionally, in the probe trial task of the MWM, compared to the AD mice, both the time and distance spent in the platform quadrant, and the number of entries into the platform significantly decreased in the AA group, while these indexes and entries into the platform quadrant were significantly increased in the MA group (*p* < 0.05 or *p* < 0.01, Figure 6C–F). The tracking plots of the MA group were evidently concentrated in the targeted platform quadrant compared with that of the AD groups and AA group (*p* < 0.05 Figure 6G). In brief, these NORT and MWM findings demonstrated that AA led to a decline in both the non-spatial and spatial memory abilities, while MA improved these cognitive functions in the AD mice through microbiota transplantation.

### 3.8. FMT Intervention Regulated SCFAs and the Relative Receptors, Brain Inflammation, and Memory Indices

**Figure 7:**
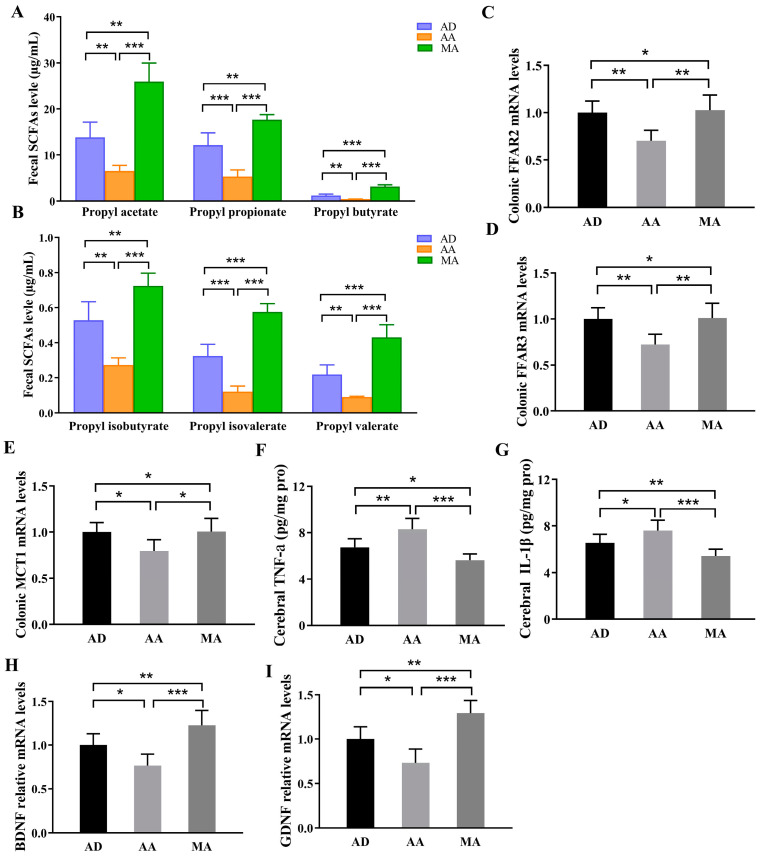
Fecal microbiota transplantation experiment demonstrated the improvement via MR of SCFAs, free fatty acid receptors, and SCFA transporters of gut, as well as learning and memory indexes and inflammatory factor of brain. (**A**,**B**) The concentrations of several types of SCFAs. (**C**,**D**) Free fatty acid receptors, FFAR2 and FFAR3. (**E**) SCFA transporter—monocarboxylate transporter (MCT1). (**F**,**G**) Inflammatory factors TNF-a and IL-1β in the brain. (**H**,**I**) The mRNA levels of learning and memory indicators in the hippocampus: brain-derived neurotrophic factor (BDNF) and glial cell line-derived neurotrophic factor (GDNF). Values are presented as means ± SEMs (n = 6 per group). *, *p* < 0.05; **, *p* < 0.01; ***, *p* < 0.001.

The FMT experiment aimed to further elucidate the mechanism of the gut–brain axis in modulating AD through MR intervention. As shown in Figure 7, compared with the AD group (approximately four-month-old AD mice), the AA group (four-month-old AD mice transplanted with fecal microbiota from 6.5-month-old AD mice of the first experiment for 8 weeks) experienced a significant decline in the levels of propyl acetate, propyl propionate, propyl butyrate, propyl isobutyrate, propyl isovalerate. The AD group (approximately four-month-old AD mice) and the AA group (four-month-old AD mice were transplanted with fecal microbiota from 6.5-month-old AD mice of the first experiment for a duration of 8 weeks) experienced a significant decline in the levels of propyl acetate, propyl propionate, propyl butyrate, propyl isobutyrate, propyl isovalerate, and propyl valerate (*p* < 0.01 or *p* < 0.001, Figure 7A,B); compared to the AD group and AA group, the MA group (four-month-old AD mice were transplanted with fecal microbiota from 6.5-month-old AD mice with an MR diet of the first experiment for a duration of 8 weeks) significantly increased these SCFAs (*p* < 0.05 or *p* < 0.01, Figure 7A,B). The administration of MR mitigated the AD-induced decline in mRNA expression of the free fatty acid receptors, FFAR2 and FFAR3, and SCFA transporter, MCT1 (*p* < 0.05 or *p* < 0.01, Figure 7C–E). FMT is one of the most direct methods for altering the composition of the gut microbiome. These FMT findings present compelling evidence that regarding the young AD mice which received the gut microbiota from older AD mice with cognitive impairment, their concentrations and related markers were reduced. However, when young AD mice were exposed to the gut microbiota from MR-treated intervention mice, their SCFA levels and related markers improved.

Additionally, in the FMT experiment, AA aggravated the brain inflammation of AD, while MA relieved the AD-induced inflammation through reducing the levels of inflammatory factors TNF-a and IL-1β in the brain (*p* < 0.05 and *p* < 0.01, respectively, Figure 7G). Meanwhile, AA decreased the learning and memory indicators BDNF and GDNF in the hippocampus (*p* < 0.05) while MA significantly increased their gene expression levels compared with the AD group (*p* < 0.01, Figure 7H,I).

## 4. Conclusions

AD is an irreversible and degenerative neurological disorder. MR has been shown to enhance learning and memory capabilities in middle-aged mice, D-galactose-induced aging mice, and AD mice [6,7,8]. The regulatory mechanisms through which MR influences AD are mainly related to its effects on brain oxidative stress. Given the complexity of AD pathology, further research is essential to fully elucidate the underlying regulatory mechanisms through which MR affects cognitive function. This study demonstrates that both a methionine-restricted diet and FMT from MR-treated donors improved the learning and memory abilities in Alzheimer’s disease through the modulation of short-chain fatty acids.

AD and dietary MR have a significant impact on the gut microbiota. Compared with cognitively healthy controls, AD patients show an increase in *Firmicutes*, *Proteobacteria*, *Actinobacteria*, and *Verrucomicrobia*, while *Bacteroidetes* are reduced in their feces [43]. In mice, although changes in *Firmicutes and Bacteroidetes* have been reported inconsistently, the reduction in *Proteobacteria* and *Verrucobacteria* was a common feature in 9-month-old and 12-month-old APP/PS1 mice [23,24]. At the genus level, compared with the WT group, AD mice experienced a distinct increase in the abundance of *Anaerotruncus, Helicobacter*, and *unclassified Erysipelotrichaceae* (*p* < 0.01, Figure 1F), which is consistent with previous reports [26,27]. *Erysipelotrichaceae* was reported as an inflammatory or Aβ-associated bacteria [44,45]. Clinical studies have revealed that individuals infected with *Helicobacter pylori* exhibit symptoms of AD, as *Helicobacter pylori* can cross the blood–brain barrier, which contributes to amyloid deposition [46]. Furthermore, the AD group showed a significant drop in the abundance of *Alloprevotell*, *Blauti*, *Roseburia*, *uncultured Muribaculaceae_bacterium*, *unclassified Bacteroidales*, or *unclassified Christensenellacea*, while MR reversed these variations (*p* < 0.05, Figure 1F). The previous literature documented the reduction of *Alloprevotel, Blautia, Roseburia*, and *Bacteroidales* abundances induced by AD [23,26,28]. *Alloprevotella* has been reported to positively correlate with antioxidant enzyme activity and negatively correlate with inflammatory factors in AD [21]. *Roseburia*, *Bacteroidales*, and *Christensenellaceae* can produce SCFAs, particularly butyrate [28,37,38]. *Muribaculaceae bacterium* is related to the intestinal mucosal immune system and has the function of promoting intestinal metabolism, and *Blautia* could alleviate inflammatory diseases and metabolic diseases [47]. Hence, these results indicate that AD induces the dysregulation of the intestinal flora; the administration of MR resulted in an amelioration of the gut microbiota, which contributes to the improvement of the mucosal barrier function and an elevation in the concentration of SCFAs.

Numerous studies have indicated a reduction in SCFA levels in AD patients [48,49]. SCFAs can affect the CNS and cognitive behavior through various molecular mechanisms, including anti-inflammatory properties [50], the induction of enteroendocrine signaling [50], the inhibition of histone deacetylase [50], the promotion of astrocyte–neuron glutamate–glutamine cycling [51], the maintenance of microglial structural and functional integrity [52], and the modulation of lymphocyte activity in the peripheral immune system [53]. SCFAs can modulate brain function by directly or indirectly influencing the gut–brain axis through immune, endocrine, vagal, and other humoral pathways [53]. Moreover, regulating the excessive activation of microglial cells in AD and promoting their transformation from M1 to M2 is beneficial for inhibiting inflammation, protecting neurons, and clearing Aβ aggregates [12]. The active SCFAs produced by microbial fermentation can alleviate inflammation, behavioral disorders, and Aβ accumulation in AD mice by regulating the activation and transformation of microglial cells [9,12,13]. As a result, SCFAs are recognized as crucial mediators of microbiota–gut–brain crosstalk. In this study, the potential mechanism believed to mainly contribute to the effectiveness of MR diet intervention or fecal transplants from MR mouse donors in improving the cognition and inflammatory biomarkers is the elevation of SCFA levels. However, based on the results of metabolomics (Figure 4), it is evident that numerous regulatory mechanisms remain to be further elucidated.

Dietary intervention, as a non-pharmacological treatment approach, not only provides assistance at the physiological level but also serves as part of overall patient health management. In the clinical research, MR dietary intervention has shown significant improvement in obese people, the elderly, and patients with cancer, which were summarized in our previous review article [54]. Moreover, commercial products have already been marketed, such as the methionine-free medical food, Hominex-2 (Abbott Nutrition, Columbus, OH, USA), and the SAA-free powdered drink, XMET XCYS Maxamaid^®^ (Nutricia Norway AS, Oslo, Norway). Therefore, the application of MR in AD patients is expected to expand in the future.

FMT, as a method for restoring a healthy microbial community, has positive effects in reversing gut dysbiosis and promoting a healthy gut state. The application of FMT holds promise as a viable therapeutic approach for neurological disorders [55]. It was reported that germ-free wild-type mice which received AD feces found a deterioration of cognitive function, compared to those receiving healthy control feces [49]. However, when a young donor-derived microbiota is transplanted into aged hosts, it attenuates selective age-associated cognitive impairments [56]. In our present study, young AD mice that receive the gut microbiota from older AD mice with cognitive impairment showed worsened gut flora and cognitive function. Conversely, when young mice were exposed to the gut microbiota from intervention mice treated with MR, their gut microbiota and cognitive function were improved. These variations resulted from the donations of different flora, confirming the direct role of gut flora in cognition, although the composition of gut microbiota in FMT mice was not provided in this study. Moreover, the FMT intervention proved to be an effective therapeutic approach for modulating the gut microbiota metabolites, which demonstrates promising outcomes in improving AD.

## Data Availability

The original contributions presented in this study are included in the article/Supplementary Materials. Further inquiries can be directed to the corresponding author.

## Supplementary Materials

The following supporting information can be downloaded at: https://www.mdpi.com/article/10.3390/foods14010101/s1.
