# Supplementary material for: Fecal Microbiota Transplantation from Methionine-Restricted Diet Mouse Donors Improves Alzheimer’s Learning and Memory Abilities Through Short-Chain Fatty Acids
Source: Foods. 2025 Jan 2;14(1):101. doi: 10.3390/foods14010101 (PMC11720665; doi:10.3390/foods14010101)
Supplement: Supplementary file 1 [file foods-14-00101-s001.zip › foods-3340401-supplementary.pdf]

Supplementary materials

Article

Fecal Microbiota Transplantation from Methionine-Restricted Diet Mouse Donors Improves Alzheimer's Learning and Memory Abilities Through Short-Chain Fatty Acids

Run Yu [1,2,†], Haimeng Zhang [1,2,†], Rui Chen [2], Yangzhuo Lin [2], Jingxuan Xu [1], Ziyang Fang [1], Yuehang Ru [1,2], Chenhan Fan [1] and Guoqing Wu [1,*]

2.3. Novel object recognition test and Morris water maze test

NORT was used to assess the non-spatial declarative memory and performed as described previously [15]. During the habituation session, mice were placed into the open field box without objects for 10 min. After 24 hours, in the training session, mice were placed in this box with two identical odorless objects for 10 min, either rectangular wooden blocks (4 × 4 ×10 cm) or hard plastic cylinders (diameter: 5 cm, height:10 cm). During the test session (24 hours after the training session), mice could explore the two objects in the same position as before for10 min, but one object identical to the original object and the other replaced by a novel object. when the mouse nose was within 2 cm to the object, it was considered to be exploring an object. Time spent exploring all the objects was collected in both the training and test session by ANY-maze Software. Moreover, discrimination of the novel object in the test session was calculated using the discrimination index (DI) [DI = (novel object exploration time - familiar object exploration time)/total exploration time].

MWM test was used to exam the spatial learning and memory and performed as described previously [16].. A round pool (diameter: 150 cm, height: 50 cm) filled with 22 ± 0.5 °C water whitened with silica or starch. The escape platform (diameter:10 cm, height: 30 cm) was movable and could move to each trial. The maze was divided into four equally spaced quadrants (NE, SE, NW, SW). The MWM included three tasks: the visible platform task, the hidden platform task, and the probe trial task. (1) Visible platform task for 1 day: It was performed as a general estimate of visual acuity and motoric ability. Fill water into the pool until the platform with a flag was 1 cm above the water surface. Each mouse performed four trials. The platform was moved for each trial. The mouse was gently lowered into the pool in different quadrants and allowed to swim freely to locate the platform within 60 s. Computer tracking program is started the moment that the animal is released. The latency to locate the platform was recorded. If time is more than 60 s, the latency is 60 s. (2) Hidden platform task for 5 days: The flag was removed from the platform. The pool was filled with water until the

platform was hidden 1 cm below the surface of the water. Silica or starch was added to the pool and mixed thoroughly before every trial to make the submerged platform invisible from the surface of the water. Each mouse performed four training trials daily for four consecutive days. During the training session, when the mouse located the platform, it was left on the platform for 10 s. If the mouse could not find the platform within 60 s, then it was placed on the platform for 20 s. (3) Probe trial task for 1 day: 24hours after the last training trial, the mice underwent a probe trial session after the platform was removed, wherein they were allowed to swim for 60 s. Number of times entering the target quadrant and platform site crossovers, time and distance spent in the target quadrant and the other quadrants were record by ANYmaze Software.

2.9. Targeted Metabolomics and 16S ribosomal RNA gene sequencing

Targeted metabolomics: Targeted metabolomics of faeces sample were tested by Wuhan Metware Metabolic Biotechnology Co., LTD (China). After the faeces sample was thawed and smashed, an amount of 0.05 g of the sample was mixed with 500 μL of 70% methanol/water. The sample was vortexed for 3 min under the condition of 2500 r/min and centrifuged at 12000 r/min for 10 min at 4°C. Take 300 μL of supernatant into a new centrifuge tube and place the supernatant in -20°C refrigerator for 30 min, Then the supernatant was centrifuged again at 12000 r/min for 10 min at 4°C. After centrifugation, transfer 200 μL of supernatant through Protein Precipitation Plate for further LC-MS analysis.The sample extracts were analyzed using an LC-ESI-MS/MS system (UPLC, ExionLC AD, https://sciex.com.cn/; MS, QTRAP® 6500+ System, https://sciex.com/).

16S ribosomal RNA gene sequencing: Fecal sample DNA was extracted with the TGuide S96 Magnetic Soil /Stool DNA Kit (Tiangen Biotech (Beijing) Co., Ltd.) according to manufacturer instructions. The DNA concentration of the samples was measured with the Qubit dsDNA HS Assay Kit and Qubit 4.0 Fluorometer (Invitrogen, Thermo Fisher Scientific, Oregon, USA). The universal primer set 338F: 5'-ACTCCTACGGGAGGCAGCA-3' and 806R: 5'-GGACTACHVGGGTWTCTAAT-3' was used to amplify the V3-V4 region of 16S rRNA gene from the genomic DNA extracted from each sample. Both the forward and reverse 16S primers were tailed with sample-specific Illumina index sequences to allow for deep sequencing. The total of PCR amplicons was purified with Agencourt AMPure XP Beads (Beckman Coulter, Indianapolis, IN) and quantified using the Qubit dsDNA HS Assay Kit and Qubit 4.0 Fluorometer (Invitrogen, Thermo Fisher Scientific, Oregon, USA). After the individual quantification step, amplicons were pooled in equal amounts. For the constructed library, use Illumina novaseq 6000 (Illumina, Santiago CA, USA) for sequencing.
